# Effect of environmental factors on growth performance of Nile tilapia (*Oreochromis niloticus*)

**DOI:** 10.1007/s00484-022-02347-6

**Published:** 2022-08-31

**Authors:** Mohamed E. Abd El-Hack, Mohamed T. El-Saadony, Maha M. Nader, Heba M. Salem, Amira M. El-Tahan, Soliman M. Soliman, Asmaa F. Khafaga

**Affiliations:** 1 Poultry Department, Faculty of Agriculture, Zagazig University, Zagazig, 44511 Egypt; 2 Department of Agricultural Microbiology, Faculty of Agriculture, Zagazig University, Zagazig, 44511 Egypt; 3 Department of Poultry Diseases, Faculty of Veterinary Medicine, Cairo University, Giza, 12211 Egypt; 4 Plant Production Department, Arid Lands Cultivation Research Institute, The City of Scientific Research and Technological Applications, SRTA-City, Borg El Arab, Alexandria, Egypt; 5 Department of Internal Medicine and Infectious Diseases, Faculty of Veterinary Medicine, Cairo University, Giza, 12211 Egypt; 6 Department of Pathology, Faculty of Veterinary Medicine, Alexandria University, Alexandria, 22758 Egypt

**Keywords:** Nile tilapia, Growth efficiency, Stoking density, Water quality

## Abstract

Aquaculture is the practice of developing aquatic animals and plants under artificial environmental conditions, either in a controlled or semi-controlled environment. Due to high animal protein demand, it is one of the world’s growing food production industries. It plays a vital role in contributing to food security and lowering the unemployment rate of the world’s growing population. This review article aims to scope sight on the environmental factors that affect the growth and economic production process of Nile tilapia. Many of these factors are listed and analyzed in this review, such as stocking densities; various feed frequencies and feeding rates; water quality; water temperature; dissolved oxygen concentration; water pH degree; ammonia (NH_3_), nitrite (NO_2_), and nitrate (NO_3_) concentration; feeding regimes; feed cost; and tank culturing system of Nile tilapia. These factors can significantly alter body weight, composition, survival, behavior, feed intake, feed conversion ratio, feeding efficiency, and the health and reproduction of *Oreochromis niloticus*. Furthermore, feeding, growth, disease risks, and survival rates are all affected by water quality parameters. In general, higher growth performance of *O. niloticus* in aquaculture can be obtained by keeping the optimum quantity of feed with proper feeding rate and frequency, maintaining a good proportion of stocking density, and regularly evaluating water quality. This review article highlights—in details—the impact of various environmental factors on growth performance criteria of Nile tilapia (*Oreochromis niloticus*).

## Introduction

In 2014, the world’s per capita supply of fish reached a new record high of 20 kg; strong aquaculture growth provides half of all fish for human consumption (FAO 2016). The aquaculture value in Africa increased by 56% from 2003 to 2007, while production increased by 100% in volume. This growth was attributed to the aquatic commodity costs, the emergence and distribution of small- and medium-sized enterprises, and the substantial investment in cage culture, followed by larger commercial enterprises (FAO 2010). UNDPI (2010) reported that, in 2007, about 28% of fish stocks monitored by FAO were overexploited, either depleted or recovering from depletion, and thus yielding less than their maximum potential owing to excess fishing pressure. A further 52% of stocks were fully exploited, producing catches at or close to their maximum sustainable limits. Only 20% of stocks were moderately exploited or underexploited, possibly producing more (Abd El-Hack et al. 2016, 2021).

Before that, Casal (2006) published that capture fisheries alone cannot meet the increasing global population and demand for fish protein. Aquaculture development is growing and cage culture today plays a vital role in meeting the demand for fish in the world (Olivares 2003; Alagawany et al. 2020, 2021). Often known as aqua farming, aquaculture is the cultivation of aquatic species such as fish, crustaceans, mollusks, and aquatic plants. Freshwater and saltwater populations are included in aquaculture and can be contrasted with commercial fishing for wild-fish harvesting under regulated conditions (Naiel et al. 2020). The current review highlights the effects of environmental factors on the growth performance of Nile tilapia (*Oreochromis niloticus*).

## Tilapia fish growth efficiency in culture

The achievements of the method of farming tilapia rely on various factors and it can be very complex to determine the optimal way under certain conditions (Graaf et al. 2005). Ofori et al. (2009) said that the benefits of using all males are that when they grow over 250 g, they grow approximately 40% faster than mixed sexes. Barman and Little (2011) tested the production of Nile tilapia (*O. niloticus*) in nylon mesh net cages (hapa).

Klanian and Adame (2013) evaluated the performance of Nile tilapia (*O*. *niloticus*) fingerlings raised at hyper-intensive stocking density in a recirculated aquaculture system (RAS). Fish (2.07 ± 0.04 g) were stocked in triplicate at 400 (T1), 500 (T2), and 600 (T3) fish m^−1^. The density of stocks had no significant effect on survival. There were substantially higher growth rates of T1 and T2 than T3. Temperature affected 41% of the SGR of T1.

The effects of feeding frequency on *O. niloticus* growth and nutrient utilization of fingerlings are studied by Jegede and Olorunfemi (2013). A 58-day feeding trial was performed to examine the effects on *O. niloticus* (3.40 g ± 0.04) at different feeding frequencies, one, two, three, and four times every day, respectively, in concrete tanks with a capacity of 400L. The feeding frequency of three times a day and other feeding frequencies considerably increased (*p* < 0.05) concerning the final average weight.

Chakraborty et al. (2011) have examined the growth rates in cistern, pen, flow-through, and pond systems of mono-sexual and mixed-sex tilapia fish. It was found that the weight, length, daily weight gain (DWG), GSR, and protein content of mono-sexual tilapia were significantly higher than those of mixed-sex fish. The fish’s weight, DWG, and protein content in the pond culture were considerably greater than in the other three culture systems. Finally, with an increased feeding rate, growth efficiency and net yield were increased, and repeated feeding for the optimum results of *O. niloticus* was recommended.

## Factors that affect *O. niloticus* fish growth performance

### Different stocking densities

Small-scale fish culture has frequently struggled due to insufficient knowledge of ideals, such as fish stock densities (Osofero et al. 2009). Survival, growth, behavior, water quality, and feeding are straightly affected by stocking density (SD). In a culture system, stocking density is the concentration of fish stocked into a system (Gomes et al. 2006; De Oliveira et al. 2012). High-density tilapia cultures have proved successful, but it has been difficult to compare the results with studies conducted on low-storage tilapia because individual studies do not address problems arising from many interactive factors (Ali et al. 2006). SD is important in determining finfish aquaculture system productivity, mainly by maximizing water utilization. However, high stocking density may also limit growth and harm fish revival when physiological and spatial needs are not adequately met (Le Ruyet et al. 2008). Increasing SD generally leads to direct increases in stress conditions, leading to a decrease in the growth rate and food utilization. On the other side, fish may not form shoals at very low densities and may be unsecured (Chambel et al. 2015).

The effect of stock density on aquaculture growth, survival, and yield is known for diversifying species and seems to have different effects on production (Garr et al. 2011). Identifying the optimum stocking density for a species is essential for optimal husbandry activity and efficient management (Chambel et al. 2015). In total, SD and fish growth are very closely correlated. The optimum stocking density ensures sustainable aquaculture, providing proper feed utilization, maximum production, a sound environment, and good health. Compared to low stock density, high stock density has many negative consequences, including food and shelter competition and rapid disease outbreaks if present. So, the stocking density for the target species in aquaculture is optimized for the preferred production level (Ferdous et al. 2014).

Tilapia is a valuable species globally, but there is no sufficient understanding of its acceptable stock density that can significantly affect tilapia production and quality (Chakraborty et al. 2011). For Nile tilapia, it is recognized that the genetic quality of farmed stocks must be routinely improved and protected (Santos et al. 2013). High fish density in fiberglass tanks interrupts breeding activity and permits the growth of tilapias between males and females into a marketable size. The flow-through system enables the easy management of fish farmers’ stocks and a high degree of environmental control on parameters such as water, Do, pH, and waste which can be changed to optimize the production of *O. niloticus* (Yakubu et al. 2014).

**Fig. 1:**
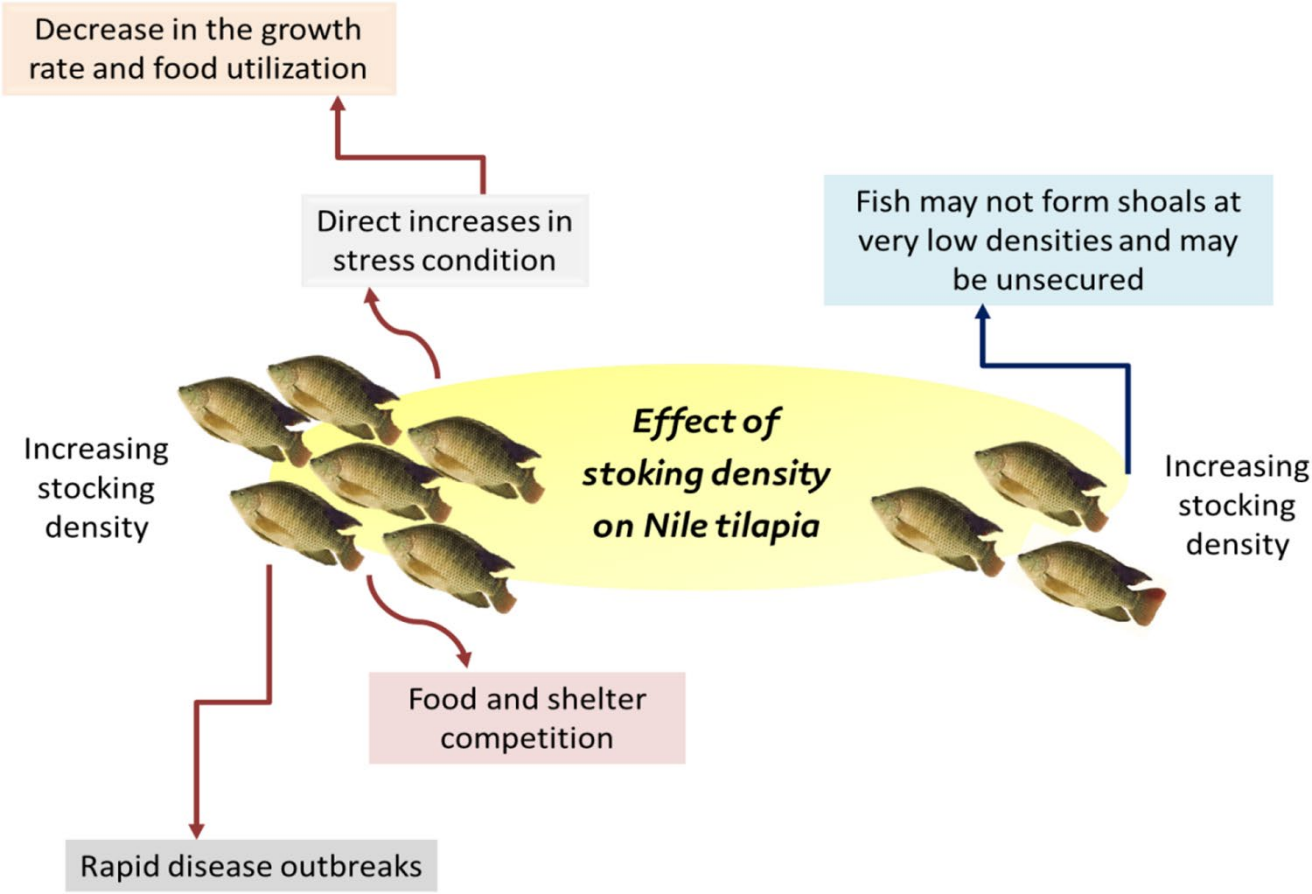
The impact of stoking density on cultured Nile tilapia

Data analysis showed significantly higher weight growth with a density of 100 m^−3^ fish, suggesting improved Nile tilapia production in circular net cages with low stocking densities. Bwanika et al. (2007) found that sex-specific differences in growth were noteworthy in *O. niloticus* where males grow much faster and larger and more standardized than females. Mainar et al. (2011) tested the viability of the cages (1 m^3^) set in farm ponds and estimated Thailand’s productivity and red tilapia subjected to varying stock densities (200, 250, 300 fish m^3^). They noticed that tilapia growth (*p* > 0.05) was not affected by the stocking density tested in the experiments. Emmanuel et al. (2013) explained that the water quality would deteriorate quickly if fish were crowded, stressed, and executed. Ali et al. (2006) reported that the ammonia level increased with increased stocking density and no water exchange. Water exchanges must be considered when reared at higher stocking densities to avoid environmental and physiological stress on fish. The impact of stocking density on cultured Nile tilapia is illustrated in Fig. 1.

### Various feed frequencies

In tilapia fish culture, it is thus important to consider the factors that influence its production such as feed type, ration size, various feeding frequencies, and how they may influence growth and feed utilization. Feeding frequency (FF) is essential for cultured organisms to achieve the highest food conversion and weight (Ferdous et al. 2014). Higher feeding frequencies decrease aggressive behavior and may result in faster growth and uniformity in size. Moreover, feeding frequency can affect the growth performance, survival, body structure (Zhou et al. 2003), and water quality (Zakes et al. 2006). Furthermore, as we know, the feed cost is one of the largest operational costs in the aquaculture industry (Ferdous et al. 2014). Proper feed management and husbandry strategies are important approaches to reducing feed costs in commercial aquaculture (Lovell 1989) and efficiently broadcasting the predetermined ratio to the culture system. Hence, the act of feeding may be pointed to as one of the most vital elements in cultural practice (Ferdous et al. 2014).

Aquaculture feeding is essential for its viability and success, like other types of farming. Feed costs are one of the major costs of aquaculture operations. The feeding practice in an aquaculture system requires choosing the appropriate ration sizes, the determination of feeding frequency and meal timing, and the successful transmission to the culture system of the predetermined ration (Anderson and De Silva 1995). The feeding frequency mainly depends on cultured species, age, size, feed quality, and environmental factors. Excellent quality feeds often fail to perform effectively unless proper feeding and feeding practices are applied. The optimal feeding rate for economic fish production should be recommended. The food system and fish growth are, in fact, very closely associated. Therefore, the feeding strategy can indicate maximum growth as feeding frequency contributes to feeding efficiency and growth response. Feeding frequency is important to ensure the cultured organism’s best FCR and weight gain (Emranul 2009).

In addition to the above, the effect of feeding frequency on the growth and production efficiency of tilapia (34.4 g) fed on a commercial diet one, two, three, or five times a day for 29 days was determined by Emranul (2009). Consumption, growth, and feed utilization were evaluated. There were no major differences in growth, feed quality, or protein use between fish fed two, three, or five times a day, but they were all significantly better than fish fed only once a day. Fish fed three meals had considerably higher gross energy and lipid and lower crude protein content than fish in other treatments (*p* < 0.05). Kaya and Bilguven (2015) investigated impacts on growth performance, feed consumption, feed conversion, and Nile tilapia’s proximate composition of four different feeder frequencies (one, two, three, or six meals a day). The average live weight used in this experiment was 9.39 ± 0.19 g. At the end of the study, it was observed that substantial differences between the groups were found to be statistically significant (*p* < 0.05) in terms of mean live weight, live weight gain, feed consumption, feed conversion ratio (FCR), and specific growth rate (SGR). Furthermore, the difference in carcass composition between the groups is statistically significant (*p* < 0.05). Feeding the correct quantity of feed correctly is very critical.

**Fig. 2:**
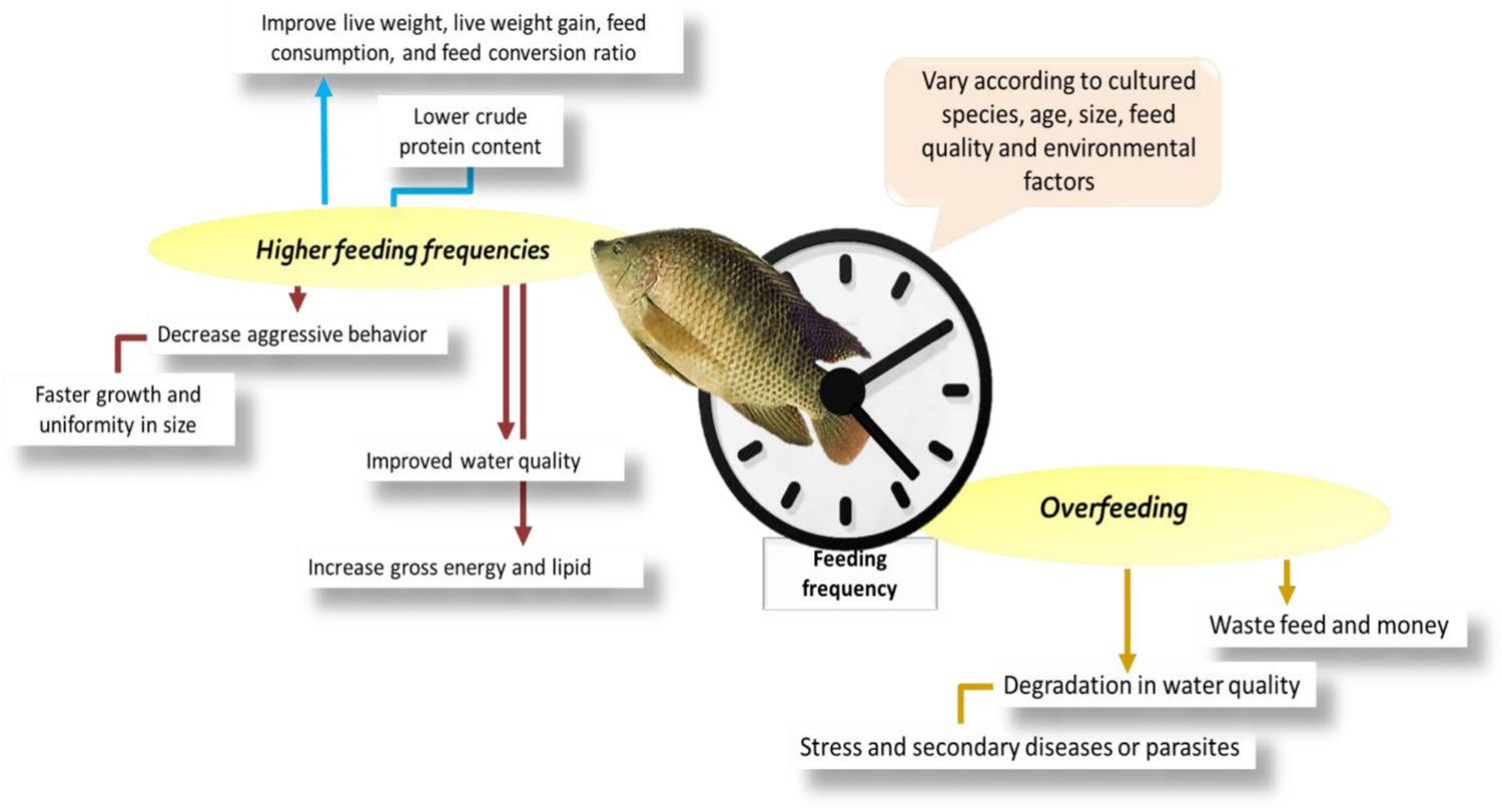
The impact of feed frequency on cultured Nile tilapia

Overfeeding wastes feed and money and can degrade water quality leading to stress and possible secondary diseases or parasites. At least 6 days a week, fish in cages should be fed. As the fish grows, the daily feed would need to be increased. During extreme overcast weather and if water temperatures reach 90°F, feeding should be discontinued (LSU 2009). Riche and Garling (2003) evidenced that, in some fish species, increased feeding rates minimize aggressive behavior. This leads to faster growth and less variation in size. However, there is a limit to the frequency, which will result in benefits. Several fish species are less efficient when fed at short intervals. Evidence suggests tilapia fed too frequently utilize feed less efficiently. It will depend on the appetite return for the optimum interval between feedings. Depending on stomach fullness, fish eat available food at times dictated by its time to empty the stomach. The velocity of emptying the stomach depends on temperature, fish weight, meal size, feed composition, and feeding frequency. The impact of feed frequency on cultured Nile tilapia is briefed in Fig. 2.

### Various feeding rates

Most wild tilapia are omnivorous, so they consume a range of things, including plants and animals, compared to many other more specialized fish. Tilapia does have unique food needs such as protein, amino acids, fats, minerals, and vitamins as other species. The nutritional requirements for fish grown in intensive recirculating systems vary from those in the wilderness. Blue-green algae and bacteria are wild tilapia grazes. They need to eat more food than farm-raised fish to meet the energy required for feeding and development. Natural food is limited in intense tank culture. Therefore, in a pelleted diet, all nutrients must be given. The improved quality and consistency of the food benefit from feeding a pelleted diet (Riche and Garling 2003). Feeding rates differ according to the water and the size of the fish. The right quantity is determined by the average body weight by a percentage. The percentage of fish weight decreases as the weight of the fish increases. The daily diet must be changed to offset growth (Riche and Garling 2003).

### Water quality

The water’s physical, chemical, and biological features play a vital role in the distribution and activity of aquatic organisms (Chaudhuri et al. 2012). The quality of water used in aquaculture influences feeding, growth, disease burden, and survival (Chainark and Boyd 2010). Water quality is regulated by complex interactions, including the weather and several other factors. For example, dissolved oxygen (DO) is connected with the production of and breathing in phytoplankton. Nitrogen waste such as ammonia is linked to the input of organic matter and ammonium excretion by fish (Sriyasak et al. 2015). De Long et al. (2009) reported that the problem is caused by poor water quality. The stresses of high ammonia, nitrite toxicity, low dissolved oxygen, high carbon dioxide, or other water quality problems can prevent fish from eating adequately. Poor water quality and lack of oxygen may lead to the loss of cultured fish (Dias et al. 2012).

Although many studies have investigated the growth, survival, and production of different tilapia species under different stocking densities, there seems to be little information about the links between water quality, such as dissolved oxygen and ammonia excretion, and the performance of growth, stock density, and size variation (Ali et al. 2006). Gorlach et al. (2013) explained that the density of bacterial species in the fish culture could be influenced by physiochemical water parameters, including pH, nutrients, and the presence of toxic compounds. At the same time, the issue of preserving sufficient water quality in fish ponds in many areas is growing (Sriyasak et al. 2014). De Long et al. (2009) explained that tilapia could withstand various environmental factors, including water quality and physical handling, threatening other organisms because of their durability.

Tools for determining the minimum amount of water needed to dissolve oxygen, temperature, pH, ammonia, nitrite, alkalinity, chloride, and calcium hardness are essential for tank growers. To accurately and regularly assess the equipment’s regular specifications, the equipment must be of a suitable standard. Generally, as De Long et al. (2009) reported, it is challenging to determine strict water quality criteria for tilapia culture. Experience at one location cannot represent the same findings as another research journal or method.

#### Water temperature

As long as their physical and biological characteristics are regulated, tilapia may develop to their full size and growth potential in many habitats (Olurin and Aderibigbe 2006). The nature of aquaculture system environments is such that water quality parameters, such as temperature, must be controlled. In fish, the temperature of the water they live in dramatically affects their physiology. Increased ambient temperatures lead to increased fish metabolic rates, leading to increased demand for food (Shackleton 2012). The temperature can also influence fish physiology, so energy consumption and metabolic demands will be affected (Byström et al. 2006).

Fish growth rates, like how to fish, and eat food, are often influenced by temperature levels. For most fish species, increases in growth rates will rise when the temperature exceeds a certain point, at which point growth declines abruptly. When it comes to growth patterns, food supply is an influential factor because of how much of it is available. On top of that, certain feeding choices limit the growth of animals, which will affect growth rates at any observed temperature (Shackleton 2012). Most fish prefer optimal temperatures for growth and survival, which can change with age and size. There are varying optimal temperatures for each early life stage, representing temporal and spatial distribution patterns. In addition, growth and development in various fish species have been studied concerning the combined effects of size and temperature (Handeland et al. 2008). Nehemia et al. (2012) described the study that the optimal temperature for tilapia growth is between 29 and 31 °C. At a temperature of 20 to 22 °C, growth is approximately 30% of its limit. On average, the lethal minimum temperature for tilapia is 10 °C or 11 °C, while stress and disease can usually strike them at 37 to 38 °C. Mirea (2013) reported that Nile tilapia (*O. niloticus*) with an average weight of 33.5 g were used to study the effect of different temperatures on growth performance, survival rate, and biochemical parameters. They were stocked in 12 rearing units at 20, 24, 30, and 28 °C (control) water temperature for 30 days.

Results showed that growth performance was not significantly (*p* > 0.05) decreased at 20 and 24 °C. The survival rate was the same for the treatments. The feed conversion ratio for fish increased with the temperature, but the difference between the high temperatures (28 and 30 °C) was insignificant. Results showed that the thermal range of 20–30 °C was suitable for intensive culture of Nile tilapia regarding the optimum growth performance and survival rate. Mjoun et al. (2010) found that the optimum temperature range for tilapia is between 22 and 29 °C; spawning generally occurs at temperatures above 22 °C. Water can retain a particular amount of dissolved gas (i.e., DO and other gases).

When water’s temperature decreases, the capacity of water to keep oxygen molecules becomes much less (Kreger 2004). Scientists performed laboratory experiments to test the development of juvenile Nile tilapia. Four thermal regimes were checked, 22, 26, 30, and 34 °C. Temperature had a noticeable (*p* < 0.05) effect on development. The weight at 26 °C and 30 °C was found to be substantially higher than the weight at 22 °C and 34 °C. DWG was better at 26 °C and 30 °C, while FCR was better at both 26 °C and 30 °C. We found that the survival rates were the same at all temperatures. This indicates that *O. niloticus’s* optimum growth and feed use occur at 26 °C and 30 °C (Azaza et al. 2008).

#### Dissolved oxygen concentration

The low dissolved oxygen concentration (DO) is associated with stress, poor appetite and slow growth, disease susceptibility, and mortality. We believe that the pond culture minimum daily DO concentration is of greatest importance. As the minimum DO concentrations decreased, growth rates decreased and became increasingly less. Concentrations should not fall below 1 mg/L in tilapia ponds (Boyd 2010).

In general, tilapias tolerate low DO concentrations even down to 0.1 mg L^−1^, but maximum growth is achieved with DO concentrations greater than 3 mg L^−1^. Oxygen is essential for fish growth and survival, and as a result, it affects fish respiration and nitrate and ammonia toxicity. Under tilapia species, the minimum DO requirement is 5 mg L^−1^, and respiration and feeding activities decrease when the DO concentration decreases (Mallya 2007).

Additionally, fish cannot use the food they eat when DO is low (Nehemia et al. 2012). Since fish are sensitive to water quality, low water quality can affect them. Feeding should be decreased or stopped when water quality drops below certain thresholds. DO levels fall shortly after feeding. Keeping DO levels above 5 ppm is best to ensure good growth. At 3–5 ppm, feeding should be decreased and discontinued at 3 ppm or lower (Riche and Garling 2003). De Long et al. (2009) mentioned that, at levels between 5 and 7 mg/L, dissolved oxygen should be in the range for tilapia tanks. DO concentrations of less than 3.5 mg/L will reduce growth and feed conversion. Short-term exposure (less than 10 min) to DO concentrations as low as 0.8 mg/L is possible with survival and recovery. Sriyasak et al. (2015) advised using aeration and mechanical mixing interventions when DO concentrations are low, which helps minimize stress on fish and prevent fish death from the accumulation of DO.

#### Water pH degree

The pH of natural water is highly affected by various variables, including the carbonate system, rock type, soil, and the contaminants discharged into the water. The primary effect on the pH of clean water is the number of carbonates (CO_42_, HCO^3−^) and carbon dioxide (Co_2_). Conversely, lower pH contains acidic water (low pH) (Kreger 2004). Another effect of pH alteration in the aquatic environment is modifying the concentration of dissolved phosphate, nitrate, and organic material used by primary producers (plants and algae).

The addition of the right mixture of inorganic and organic molecules would result in cascading impacts for all organisms in the system, which will decrease plant yield (Amico 2000). White et al. (2014) recorded that animal physiology works in these species-specific environmental conditions. Effects on fish survival and performance can occur when the water pH deviates from the ideal range for the species. When exposed to challenging pH conditions, fish change their behavior and physiology. Additionally, the water’s hydrogen ion concentration (PH) and ammonia levels are critical for tilapia culture. 

El-Sherif and El-Feky (2009) studied the performance of Nile tilapia (*O. niloticus*) fingerlings at different pH levels (6, 7, 8, and 9). Growth efficiency was significantly (*p* < 0.05) reduced at pH 6 and 9, except at pH 7 and 8. During the whole trial, there was no death. At pH 6, the FCR increased due to a substantial increase (*p* < 0.05) in its value. Even though freshwater fish can adapt to stressful water pH, raising them in these conditions should be avoided (Reboucas et al. 2015). According to El-Sherif and El-Feky (2009), rearing Nile tilapia should have pH levels between 7 and 8. Nobre et al.’s (2014) findings conflict with El-Sherif and El-Feky (2009), who discovered the optimal pH range for rising Nile tilapia juveniles in green water is 5 to 8.

Rising or decreasing water pH has no major effect on the diversity or abundance of organisms in aquatic environments. However, a major shift in pH can significantly alter the diversity and composition of organisms in freshwater systems since fewer species can survive such harsh environmental conditions. In particular, fish are far more susceptible to changes in pH concentration (Salih 2007).

#### Ammonia (NH_3_), nitrite (NO_2_), and nitrate (NO_3_) concentrations

Ammonia is a dissolved gas in natural waters such as surface, wastewater, and some well waters. It is the largest nitrogenous waste product of fish, resulting from organic matter decomposition. Mostly removed by plants or bacteria, it is soluble in water, particularly at low pH levels (as a nutrient or energy source). Un-ionized ammonia (NH_3_) and the ionized form (NH^4+^) are found in water in varying amounts depending on pH and temperature. As pH increases, the proportion of un-ionized ammonia increases, which is toxic to fish (Stone and Thomforde 2004). Ammonia and carbon dioxide are also liberated into the water column during phytoplankton busts.

Because of the low buffering effect of freshwater, carbon dioxide will accumulate in ponds, lowering the pH, reducing the amount of un-ionized ammonia, and decreasing the buffering capacity. The carbonate alkalinity of marine fishponds buffers their effect, which allows for higher levels of un-ionized ammonia, which is toxic. With pH 7, less than 1% of the total ammonia is in the toxic un-ionized form, at pH 8, between 5 and 9%, and at pH 9, approximately 30–50%. But, at pH 10, about 80–90% of the total ammonia is in the toxic un-ionized form. As low as 0.2 mg/L, prolonged exposure to toxic ammonia starts the first death, while un-ionized ammonia causes a tilapia to lose its appetite at concentrations as low as 0.08 mg/L (Nehemia et al. 2012).

El-Sherif and El-Feky (2008) cited that, at concentrations of 7.1 mg/L, ammonia is toxic to tilapia, while at concentrations as low as 0.1 mg/L, it has the opposite effect. It is calculated that the optimal concentrations are less than 0.05 mg/L. Morrow (2009) studied the growth and respiratory efficiency of juvenile Nile tilapia subjected to elevated (sub‐lethal) and low levels of total water ammonia (TAmm), and it was found that high levels of TAmm (1000, 2000, and 4000 μM) decreased oxygen consumption and ventilation rates, and caused significant impairment to tilapia growth. In addition, TAmm (with a concentration of ≤ 300 μM) does not appear to affect growth. In most cases, warm-water fish are more tolerant to ammonia toxicity than their cold-water counterparts (Timmons et al. 2002). Sriyasak et al. (2015) write that ammonia has high acute toxicity that originates from its effects on the central nervous system; 7.40 mg/L of ammonia killed all the tilapia fingerlings in 24 h. At harmful amounts of ammonia, fish cannot excrete ammonia, so it accumulates in the blood and tissues, altering enzyme function.

Feed conversion is weaker, growth rates are slower, and disease resistance is decreased (Gandhi 2012). At higher temperatures and pH values, ammonia is more harmful to marine organisms. Increasing the pH means increasing the volume of un-ionized ammonia. The NH_3_ to NH_4_ ratio increases by 10 for each 1-unit increase in pH and 2 for each 10-unit increase in temperature (Levit 2010). WWI (2009) stated that fish farms are often sources of water contamination, particularly those that raise carnivorous fish. These farms are associated with elevated nitrogen and nutrients, which can cause harmful algal blooms and dead zones.

**Fig. 3:**
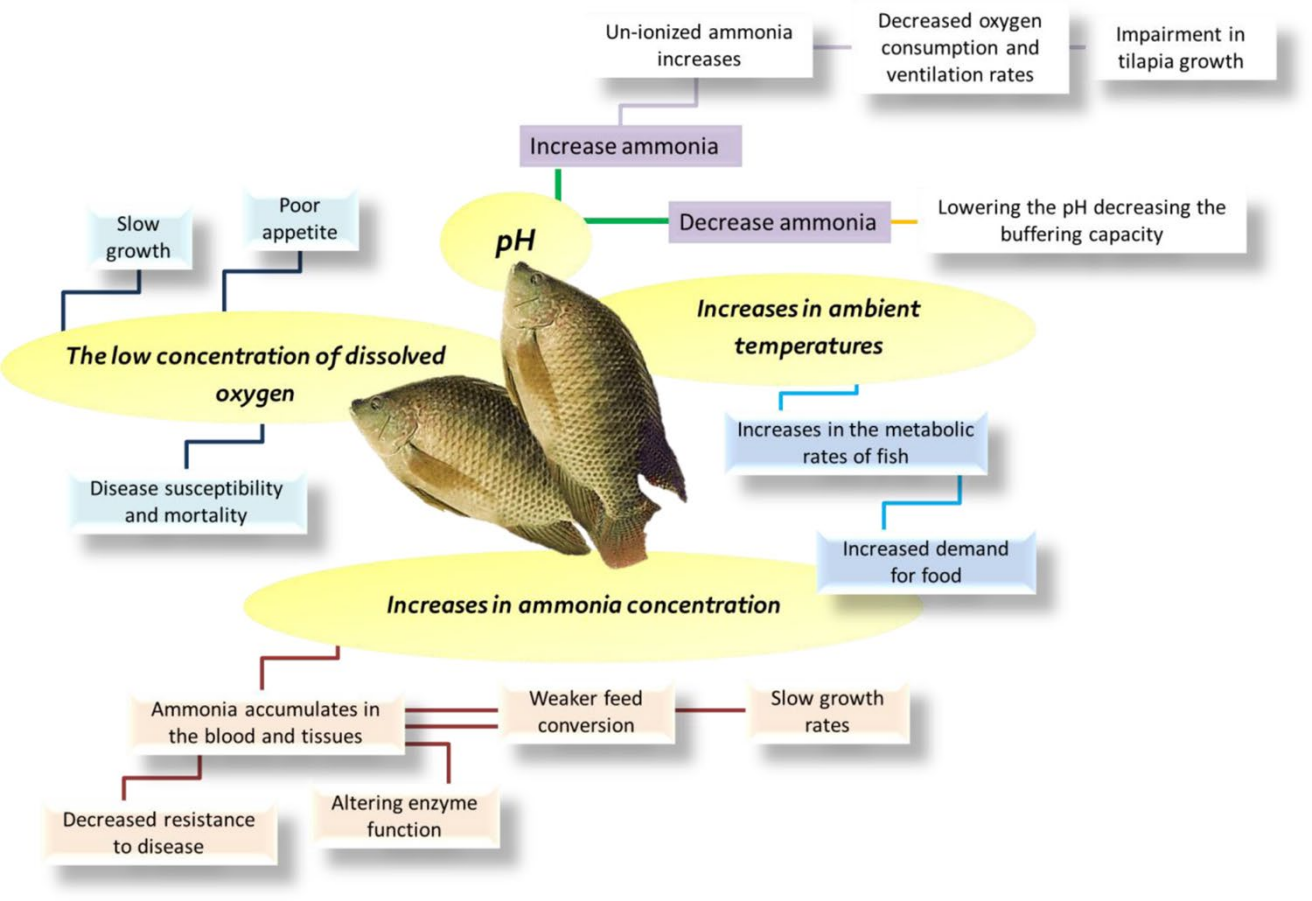
The impact of water quality parameters on cultured Nile tilapia

In commercial fish farming, since higher profits can be obtained by keeping fish in greater densities, antibiotics and other treatments for diseases end up in the water. In fish culture systems, ammonia is excreted as waste into the water, after which nitrogen is released as nitrite. In general, ammonia nitrogen produces both ammonia (NH_3_) and ammonia nitrogen (NH_4_). After the ammonia has been formed, both ammonia and ammonia nitrogen are converted to nitrite (NO_2_). The work of Ali et al. (2006) is focused on the effects of stocking density (10, 15, 50, and 75 fish in 65-L tanks) and ammonia excretion on Nile tilapia (*O. niloticus*) weighting 12.19 ± 1.21 g. The experiment proved that, with a stocking density of 15 fish/tank (2.81 g fish/L), raising the amount of *O. niloticus* from 2.81 g/L to 75 fish/tank (14.07 g fish/L) increased the ammonia level (1.48 to 26.44 mg/L) and yielded lower growth rates and better feed conversion ratios for fish reared at lower (15 fish/tank) densities. The impact of water quality parameters on cultured Nile tilapia is briefed in Fig. 3.

## Feed and feeding

Commercial profitability depends on consumer demand and production cost. Most of the production cost is in the feed (Daudpota et al. 2016). Nutrition is an important factor influencing fish efficiency, particularly in aquaculture. It is affected by fish behavior, stocking density, feed quality, daily ration size, feeding frequency, and water temperature (Alemayehu and Getahun 2017). More knowledge of the ideal feeding scheme is required to monitor feed intake, growth, and chemical composition and minimize water quality issues due to excessive feeding (Ertan et al. 2015).

Following proper stocking, the most important element of fish culture is offering quality feed to the fish in the proper quantities. The diet should contain all necessary nutrients, including vitamins and minerals. Tilapia can best be fed on commercial pellet diets. The amount of protein in fish should be approximately 32 to 36% for 1 to 25 g of tilapia and 28 to 32% for larger fish. Nutrition and feeding are the most significant production costs (McGinty and Rakocy 2005). Feeding above the optimum amount wastes both money and food, with the possibility of water quality degradation resulting in stress and the creation of secondary diseases or parasites. Feeding the animals inappropriately will reduce their growth, production, and benefit (LSU 2009). On the same side, Daudpota et al (2016) cited that overfeeding fish overloads the stomach and intestine, resulting in reduced digestion efficiency and feed efficiency. Thus, the amount of food fed each time, or feeding frequency, can influence how much food is used. This is because food not consumed directly is dissolved in water and then allowed to undergo lixiviation. Feed conversion ratio increase and environmental pollution are the results. Because fish can acquire high amounts of nutrients from their diet, frequent diet administration is sufficient for fish juveniles.

Sriyasak et al. (2015) cited that fishermen or fishers should pay attention to reducing the number of excess nutrients or managing sediments to maintain pond water quality and ecosystem stability. Feeding errors, which are fairly common in fish culture, include feeding with low-quality feed, insufficient feed, feeding incorrectly, overfeeding, and feeding at the wrong time of the day. There are no easy solutions for many of these problems, and there will be a degree of stress. Typically, the goal of management is to lower the overall stress imposed on the fish from handling and feeding activities (LSU 2009). Lowering the feed conversion ratio ensures better-quality fish.

To meet the optimal cage growth conditions, Africa’s aquaculture FCRs use tilapia systems that vary between 1.4 and 2.5 FCR. Exceeding the industry standards could cause a high amount of “fines” (feed dust) in the feed significant variation in the reported nutrient content, or an excess of unrecorded mortality (Ofori et al. 2009). One of the characteristics of tilapia being well-suited for simple hatchery development is that new fry does not require specialized live feeds such as rotifers, microalgae, or *Artemia*. They can be given commercial dry feeds (De Long et al. 2009).

## Feed and feeding cost

In aquaculture and fish culture, the major factors for limiting fish production are fish nutrients, dissolved oxygen, pH, Co_2_, N_2_, and waste product accumulation (Alamin et al. 2017). Feed accounts for more than half of the production cost in aquaculture (FAO 2009). The bulk of variable costs for fish farms can be attributed to feeding, which constitutes around 50 to 70% of the overall variable costs. De Silva and Hasan (2007) mention that feed costs usually account for 40–60% of production costs in semi-intensive and intensive aquaculture systems. Some farmers have started using alternative feed due to the increasing cost of commercial tilapia feed. The commercial feed may be added to kitchen and restaurant waste or chicken byproducts to rotate it. Tilapia feed is often supplemented with cheaper chicken or duck feed. Tilapia feed pellets made on the farm are another investigated solution (Ofori et al. 2009). Currently, a species-specific and full diet can be constructed for commercial species.

Commercially available diets are appropriate for both health and growth. A nutritionally complete diet should be fed to sustain fish at optimum levels, with the proper protein and energy types. It must be supplemented with a complete array of essential and non-essential vitamins and minerals. Many commercial feed mills can also produce both full and supplementary diets. Since a species-specific diet is required, the fish farmer must purchase a complete diet (LSU 2009). Feeding rates are more likely to be measured by fish culturists. Feed quantities are generally measured in two ways. One method relies on feed conversion measurements and adjusts feeding rates weekly to estimate growth. The second approach uses data on a fish sample from the tank and changes the feeding amount built on this study (LSU 2009).

Fish eat the most actively and intensely at their desired and optimal temperature and when oxygen levels are high. When water temperature increases, fish prefer late afternoon (and warmer) water during the spring but prefer early morning (and cooler) water during the summer.

These fingerlings usually consume around 4 to 5% of their body weight. As they grow to larger fingerling size, the rate will fall to 3%. The approaching harvest size will decrease to 2% or less. Local grasses and vegetation should be used to feed fish, which have anti-parasitic or disease-suppressing benefits. Feed is typically a byproduct of daily activities: banana peels, cassava leaves, duckweed, rice bran, maize leaves, etc. When fish are young, chop small pieces of grass and vegetation for them to eat (IIRR et al. 2001). Soltan (2016) reported that although sinking pellets have proven effective, special care must be taken to prevent the pellets from being wasted. Disintegrating shot pellets have a greater tendency to drift in the water. McGinty and Rakocy (2005) write that it is important to feed tilapia more than once daily; feeding tilapia in a single feeding cannot meet their daily requirements for growth. Feed a fish weighing between 25 and 50 g three times a day.

Ofori et al. (2009) reported that the crude protein content of pelleted fish fed to cages should be approximately 28–32%. To be optimal, a lower protein diet should be provided to smaller fish, but they are not yet available in the region. In many cases, fish were fed at a declining rate of 10%, down to 1% of the estimated average body weight based on the weekly or monthly average weight. On average, fish food should be provided to fish in 2–3 feedings, manually using floating or sinking feed. Feeder floating is typically more costly, but it allows for feed tracking. The preparation costs for floating pellets are considerably higher than for sinking pellets. A tray should be inserted into the cage and the pellets can be poured into the tray if sinking pellets are to be used (Soltan 2016).

## Tilapia fishes’ culture in tanks

There are some benefits of intensive tank culture instead of pond culture. High fish density in tanks disturbs breeding behavior. This enables the fish culturist to easily increase the marketable fish size by increasing them together and exerting significant environmental control over the parameters they need (Yakubu et al. 2012). De Long et al. (2009) mention that using tanks gives the fish culturist more control over stock numbers and environmental parameters like water temperature, DO concentration, and pH, which they can change to optimize yield.

Additionally, food and harvesting operations are faster and require less labor than ponds. In small tanks, treating diseases with therapeutics added to the culture is feasible and economical. Riche and Garling (2003) write that tilapia is well-suited for culture in ponds, cages, tanks, or raceways. Tank culture is useful because it decreases time and effort in harvesting and feeding. When warm water is unavailable due to climatic conditions, indoor tank culture is preferred. De Long et al. (2009) said that tilapia fish are suitable for tank culture, and their thick slime coat resists abrasion and bacterial infections that might affect other fish.

Tilapias perform well in tank systems with consistent, good water quality but also show remarkable tolerance to variable or bad water quality. Ali et al. (2006) said that stocking density and water volume per fish are major factors determining optimum tank culture system production. Alamin et al. (2017) explained that preparing the tanks before starting to work with fish in tanks is important. Just make sure your aquarium is in the best shape possible. That is particularly important for the fish’s well-being and the aquarium’s solvability. Although Alamin et al. (2017) clarified that environmental constraints, including land use disputes, source of water, water quality, and sub-optimal temperatures, will make it difficult to grow tilapia in substitutional water sources, such as ponds, lakes, cages, and reservoirs, a greenhouse could be used to regulate temperature while mitigating the other constraints.

Alamin et al. (2017) use a green water technology (GWT) system in indoor tanks to stock Nile tilapia, rui, catla, and common carp. No artificial feed was provided from stocking to harvest. GWT culture of tilapia with Indian major or exotic carps indicates that GWT has potential profit due to high productivity, an average of 150.99 ± 0.5 g/tilapia within 120 days, and no fertilization and feeding costs. Conversely, water pumping and aeration costs can rise as the tank capacity does (De Long et al. 2009).

## Impacts of feed rates on the chemical composition of *O. niloticus*

In the study reported in 2013 by Yakubu et al. (2012), the authors investigate the influence of stocking density on survival and body composition in a semi-continuous flow-through culture system for *O. niloticus*. He found that major variations among the three stocking densities were only major in dry matter composition (DM). Khattab et al. (2004) studied the growth response and body composition of *O. niloticus* (1.8–2.5 g/fish) at two stocking densities (15 and 30 fish/100 L).

To learn about the impact of feeding frequency on growth efficiency and body composition, researchers Daudpota et al. (2016) examined the growth performance. He found that crude protein, total lipids, and ash were significantly affected when stocking density was varied. The fish were fed two, three, four, and five times daily. Feed frequency of four to five times daily showed significantly higher weight gain, specific growth rate, and feed conversion ratio. Feeding frequency did not affect the entire body’s moisture, protein, or ash content. Feeding four or five times a day greatly improved the lipid content of the fish. El-Saidy and Gaber (2005) examined the effect of three feeding levels (1%, 2%, and 3% body weight (BW) day-1) on growth performance and body composition of *O. niloticus* with an average initial weight of 61.9 ± 6.03 g per fish in concrete tanks. The study showed that the growth rate increased with rising feeding levels. Additionally, this study observed a similar pattern in mean BW (g), real growth rate (%per day), feed conversion ratio, and survival rate (%). The diet level did not significantly affect whole fish fat and energy contents (*p* > 0.05). Feeding frequency greatly affected the level of protein and ash.

## Conclusion and recommendations

Aquaculture involves cultivating fresh and saltwater populations under controlled or semi-controlled environmental conditions. Because of its high-density stocking ratio, low production cost, ability to occupy different trophic levels, disease resistance, low dissolved oxygen tolerance, high ammonia concentration, and ease of reproduction in captivity, Nile tilapia can be chosen as a good candidate for aquaculture. Nile tilapia growth performance is highly affected by managerial factors such as stocking density, food quality, culturing system, feeding frequency, and rate and water quality parameters such as water dissolved oxygen, salinity, water temperature, pH, and ammonia, nitrite (NO_2_) and nitrate (NO_3_) concentrations. It was recommended to keep ideal stocking densities, feeding frequencies and rates, and feed quality, and furthermore regular evaluation of water quality parameters under different culturing systems.

## Data Availability

Not applicable.
